# Assessment of non-financial incentives for volunteer community health workers – the case of Wukro district, Tigray, Ethiopia

**DOI:** 10.1186/1478-4491-12-54

**Published:** 2014-09-22

**Authors:** Fisaha Haile, Dejen Yemane, Azeb Gebreslassie

**Affiliations:** College of Health Sciences, Department of Public Health, Mekelle University, Mekelle, 1871 Ethiopia

**Keywords:** Non-financial incentives, Motivation, Volunteerism

## Abstract

**Background:**

Volunteer community health workers (VCHW) are health care providers who are trained but do not have any professional certification. They are intended to fill the gap for unmet curative, preventative, and health promotion health needs of communities. This study aims to investigate the non-financial incentives for VCHWs and factors affecting their motivation.

**Methods:**

A cross-sectional quantitative study was performed from February to March 2013. A total of 400 randomly selected female VCHWs were included using the district health office registers. Finally, multivariate logistic regression was used to determine the independent predictors of VCHW motivation.

**Results:**

Significant numbers (48%) of study participants have mentioned future training as a major non-financial incentive. Age between 20 and 36 years old (adjusted odds ratio (AOR) = 1.45, 95% CI = 1.18, 2.13), married VCHWs (AOR = 3.84, 95% CI = 1.73, 5.02), presence of children under five years old (AOR = 0.2, 95% CI = 0.09, 0.71), allowing volunteer withdrawal (AOR = 1.35, 95% CI = 1.06, 2.47), and establishment of a local endowment fund for community health workers after they left volunteerism (AOR = 1.11, 95% CI = 1.05, 1.91) are all factors associated with VCHW motivation.

**Conclusions:**

Future training was mentioned as the prime non-financial incentive. Age, marital status, presence of children under five, allowing volunteer withdrawal, and establishment of a local endowment fund were identified as the independent predictors of motivation. Therefore, considering a non-financial incentive package, including further training and allowing volunteer withdrawal, would be helpful to sustain volunteerism.

## Background

Volunteer community health workers (VCHWs) are health care providers who are trained but do not have any professional certification. They can deliver a variety of community-based health care services, and are particularly important in areas where the use of facility-based services is low [1]. Moreover, it was intended that community health lay workers would fill the gap for unmet curative, preventative, and health promotion health needs of communities [2]. Generally, VCHWs can increase access to health services and have played a part in primary health care in areas such as maternal and child health care service programs. VCHWs received less attention in the 1990s, but are now again at the center of discussions about how to improve coverage and equity, particularly in populations with limited access to health facilities [3].

VCHWs have been promoted in the implementation of packages of interventions aimed at reducing neonatal mortality, such as antenatal home visits, promotion of immediate and exclusive breastfeeding, skin-to-skin care, appropriate care of the skin and umbilical stump, and recognition of sick newborns and treatment with antibiotics [3, 4]. Delivery of interventions in the home by VCHWs is viewed as critical during the first month of life, when many families observe a period of postpartum confinement making them less likely to seek care or advice from outside the home. VCHWs have been effective in tracking pregnant women through the postnatal period and in raising awareness of appropriate maternal and newborn care practices [5].

VCHWs work exclusively in the community settings and serve as connectors between health care consumers and providers to promote health among groups that have traditionally lacked access to adequate care. By identifying community problems, developing innovative solutions, and translating them into practice, community health workers can respond creatively to local needs [6]. Consequently, there are individual, family, community, and organizational factors that affect VCHW motivation. At the individual level, VCHWs are predisposed to volunteer work and apply knowledge gained to their own problems and those of their families and communities. Families and communities supplement other sources of motivation by providing moral, financial, and material support, including service fees, supplies, money for transportation, and help with farm work and VCHW tasks [7]. Nevertheless, even when monetary or in-kind incentives are provided to VCHWs, they are not sufficient to maintain and retain their motivation. Other types of incentives, often intangible, are critical to job satisfaction and fulfillment. These incentives include a good relationship with the community, personal growth and development opportunities, training, and other non-financial support. Perhaps the most important non-monetary incentive, a good relationship with the community, is above all the motivating factors [8].

The VCHWs are the only unpaid health workers in the Ethiopian health care system. Therefore, identifying the non-financial incentives is very crucial and timely. Motivation to become VCHW appeared to originate from desire for self-development, improve community health, and utilization of free time; thus, this study aims to investigate the non-financial incentives and factors that affect motivation of VCHWs.

## Methods and materials

### Study design and setting

This community-based cross-sectional study was performed from February to March 2013, in Kilte Awlaelo district, Tigray regional state, located 829 km north of Addis Ababa. Based on the projection of the 1994 census, in 2008 the total population of the district was 157,500 [9], with a total of 24,615 households in the district.

The Health Extension Program was launched by the Government of Ethiopia in 2005 to improve access to basic health services to the rural population. At the local level, the implementation of the Health Extension Program is based on the construction of health posts and the deployment of female Health Extension Workers (HEWs). HEWs have received one-year training and engage in disseminating preventive health messages and providing selected curative services. HEWs also train, mentor, support, and supervise VCHWs [10]. In Ethiopia, VCHWs are community members who are trained to become model families and who subsequently work to share health information with their communities towards achieving better health outcomes [11].

Training of VCHWs was given by HEWs at the local health post in most cases. The issues covered in the training of VCHWs generally focus on hygiene and sanitation, antenatal care, immunization, delivery care, maternal and infant nutrition, growth monitoring, family planning, and malaria. Every VCHW is typically given responsibility over five households in their community in most regions of Ethiopia and five mothers in the Tigray region, to whom they promote positive health practices through household visits [11].

### Study population and sampling techniques

All VCHWs working in Kilte Awlealo district were included. Sample size was determined with the assumption of single population proportion considering 0.5 previous proportion, 5% margin of error and 95% confidence intervals (CI) and 5% non-response rate and the final sample size was computed to be 400. In Kilte Awlealo district there are 19 kebelles (the smallest administrative unit in Ethiopia) and the population of each kebelle ranges from 4,779 to 12,595. The number of households in each kebelle ranges from 956 to 2,519 and there are on average more than 290 VCHWs per kebelle. Based on this, simple random sampling proportional to size was used to select the study subjects (VCHWs) using the health office and HEW registers.

### Data collection technique and data quality control

A structured face-to-face interview questionnaire, which includes socio-demographic and other characteristics that can measure non-financial incentives and motivation of VCHWs, was used. The questionnaire was prepared in English first and then translated in to the local language, Tigrigna, and was developed after a literature review [8, 10–14]. Ten experienced data collectors were hired to collect the data and one day training on the content and methods of data collection was given for data collectors and supervisors. Supervision was also performed on the spot by supervisors and principal investigators. Moreover, the data collection tool was pretested in 5% of the total sample size in areas that were not included in the final study. Finally, data cleaning and cross-checking was carried out before analysis.

### Data management and analysis

After checking for completeness and consistency of the data on the field, it was entered into Epi-Info 2005 version 3.5.2. Finally, data was exported to SPSS version 16 and cleaned by running frequencies. Exploratory data analysis was carried out to check the level of incompleteness and inconsistencies. Description of participant’s characteristics was performed by percentages. Additionally, the non-financial incentives mentioned by VCHWs were summarized by percentages. The outcome variable (motivation) of VCHWs was measured by ten index, equal weight questions (yes = 1/no = 0) and the above median value score of the index questions was used as the cutoff point to classify as motivated or not. Finally, percentage was used to summarize the outcome variable (motivation VCHWs). Bivariate logistic regression was carried out to see the crude relationship of each independent variable with the outcome variable (motivation of VCHWs). Variables which were found to be significant at *P* <0.05 were taken to the multivariate logistic regression to see the independent predictors of motivation of VCHWs. In the model-building procedure, a stepwise back ward regression technique was applied. Proportions and odds ratios with 95% CI were used to interpret results of the study.

### Ethical statement

Ethical approval and clearance was obtained from Mekelle University, College of Health Sciences Ethical review committee. A formal cooperation letter was obtained from Mekelle University and Tigray Regional Health Bureau.

Verbal consent was obtained from the study participants after explaining the objective, benefits and harms of the study because the participants couldn’t read and write. The right of participants to anonymity and confidentiality was ensured by excluding personal identifiers.

## Results

### Characteristics of the study participants

A total of 400 VCHWs were interviewed and the response rate was 100%. Almost all (97%) of the participants were orthodox religion followers and their mean age was 32.5 with a standard deviation of 8.3 years. Around half (49.5%) of the study participants had attended formal education. Of all VCHWs who attended formal education, 68.3% attended primary education and the rest attended secondary education and above. The average family size of the study participants was 5 with a standard deviation of 1.90 and the median household income was 175 birr with a range of 2,290. Most (60.2%) of the participants had children under five years old (Table 1). More than half (59.0%) of the VCHWs expressed that they had been nominated by the community they live and work with. A significant number (37.2%) of them responded that they joined volunteerism because of their pursuit to serve their community. Almost all (98.5%) of the respondents had participated in payment-free community services other than their current assignment. Thus, it helps for 96.5% of them to gain experience to better perform their current payment-free volunteer community health service.

**Table 1:** **Characteristics of volunteer community health workers in Kilte Awlealo, 2013 Northern Ethiopia**

Variables	Number (n = 400)	Percent
**Age**		
≤20	25	6.2
21–36	234	62.2
>36	125	31.2
**Educational status**		
Unable to read and write	201	50.3
Primary	141	35.3
Secondary	40	10
Post-secondary	18	4.5
**Average monthly income**		
≤500	324	81
501–1,000	63	15.8
≥1,001	13	3.2
**Marital status**		
Single	36	9
Married	234	58.5
Divorced	98	24.5
Widowed	32	8
**Family size**		
≤4	141	35.2
>4	259	64.8
**Number of children under 5 years old**	**N = 245**	
≤1	194	79.2
>1	51	20.8

### Non-financial incentives

**Figure 1:**
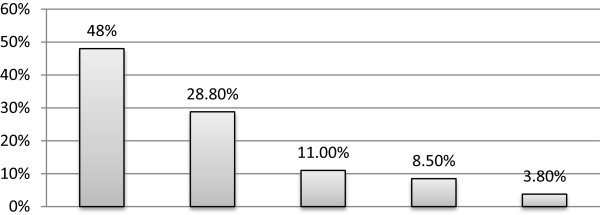
**Non-financial incentives suggested by volunteer community health workers in Kilte Awlealo, Northern Ethiopia, 2012.**

A significant number (48%) of the study participants mentioned future training as a major non-financial incentive followed by reward proposal (certificate) (28.8%; Figure 1).

Of the total VCHWs interviewed, 69.5% had their husband’s support. Most (98.5%) of the study participants agreed that it would help them to perform better if they had been selected by the community they work with. Additionally, 97.5% of study participants agreed on the importance of the establishment of community organizations that foster the task of VCHWs. Nearly all (97.5%) of the respondents reaffirmed that the community recognizes them as its own servants for the betterment of their welfare. The minimal working hour shift was also suggested by 97.8% of the participants to be a motivator since most had to deal with the running of almost all family affairs on top of their volunteerism. The average working hours with standard deviation suggested by the VCHWs was 2.30 ± 1.2 hours per day. Around 44.2% of them expressed that there was a difference in tangible incentives across VCHWs in their area. Feedback from the community was also mentioned by 96.5% of participants as an important factor to improve their performance. Inadequate, compensation, or absence of any financial or non-financial incentives was mentioned by 30.2% of participants as a reason for attrition whereas family reasons constituted about 28.5% of attrition of VCHWs.

### Factors that affect the motivation of volunteer community health workers (VCHWs)

The bivariate analysis of the relationship between motivation of VCHWs to continue their volunteerism and the presumed predictor variables age, marital status, duration of service as a VCHW, existence of children under five, husband support, allowing volunteer withdrawal, and establishment of local endowment funds were found to be significant (Table 2).

**Table 2:** **Factors that affect motivation of volunteer community health workers, 2013, Kilte Awlealo, Northern-Ethiopia, 2013**

Variable	Motivation		COR (95% CI)	AOR (95% CI)
	Yes (%)	No (%)		
**Age**				
≤20	19 (6.6)	6 (5.4)	1.55 (0.57, 4.16)	1.61 (0.47, 5.50)
21–36	185 (64.2)	65 (58.0)	1.40 (1.07, 2.21)	1.45 (1.18, 2.13)
>36	84 (29.2)	41 (36.6)	1.00	
**Marital status**				
Single	23 (8.0)	13 (11.6)	1.47 (0.69, 3.15)	1.15 (0.44, 2.19)
Married	194 (67.4)	40 (35.7)	4.03 (2.48, 6.55)	3.84 (1.73, 5.02)
separated	71 (24.7)	59 (52.7)	1.00	
**Duration stayed as a VCHW**				
≤2 yrs	258 (89.6)	96 (85.7)	0.4 (0.08, 1.5)	0.2 (0.09, 2.1)
>2 yrs	30 (10.4)	16 (14.3)	1.00	
**Presence of children under 5 years old**				
Yes	103 (35.8)	52 (46.4)	0.16 (0.07, 0.56)	0.2 (0.09, 0.71)
No	60 (53.2)	185 (64.5)	1.00	
**Husband support**				
Yes	234 (81.2)	44 (39.3)	6.70 (4.14, 10.83)	8.48 (3.6, 19.86)
No	54 (18.8)	68 (60.7)	1.00	
**Establishment of local endowment fund**				
Yes	232 (80.6)	88 (78.6)	1.13 (0.66, 1.93)	1.35 (1.06, 2.47)
No	56 (19.4)	24 (21.4)	1.00	
**Allow volunteer withdrawal**				
Yes	208 (72.2)	82 (71.4)	1.13 (0.66, 1.93)	1.11 (1.05, 1.91)
No	80 (27.8)	32 (28.6)	1.00	

In multivariate logistic regression, VCHWs who were between 20 to 36 years old age group were 1.5 times more likely to stay motivated than VCHWs >36 years old. Married women were also 4 times more likely to stay motivated than separated ones. The presence of children under five was one of the factors that affected motivation of VCHWs, i.e., VCHWs who did not have children under five were 80% times more likely to stay motivated than those who did. Allowing volunteer withdrawal and the establishment of a local endowment fund for community health workers after they left volunteerism increased the motivation of VCHWs by 1.13 and 1.19 times, respectively (Table 2).

## Discussion

Nowadays, non-financial incentives and motivation of VCHWs are at the center of sustaining volunteerism and addressing the wider scope of grass root level community health problems. However, little is known about the non-financial incentives to keep VCHWs motivated. Few studies in Ethiopia and other areas have used qualitative methods to discover non-financial incentives; however, none performed any quantification of those non-financial incentives [10].

In this study, future training is the most common non-financial incentive mentioned by almost half of the participants. Moreover, reward proposal (certificates), celebration of VCHWs day, and free medical services were mentioned by 28.8%, 11.0%, and 8.5% of the participants, respectively. This is consistent with a qualitative study performed in the Tigray region by L-10 k which reported that increased recognition in the community, future training, prizes, and employment and financial compensation in polio or other vaccination campaigns were mentioned as non-financial incentives for VCHWs to retain their volunteerism [11]. Moreover, according to a qualitative study performed in Blantyre, Malawi, feelings of empathy, altruism, and religious convictions were mentioned as intrinsic factors for volunteerism. Whereas, expected opportunities for loans to start businesses, recognition by the community, and eventual employment were mentioned as extrinsic factors that motivate VCHWs [7, 12].

Most (98.5%) of the study participants agreed that it would help them to perform better if they had been selected by the community they work with. In Bangladesh, the same procedure was followed to select VCHWs with application of certain criteria accepted by the community [8]. Additionally, in this study, 97.5% of study participants agreed on the importance of establishment of community organizations that foster the task of VCHWs.

There are various motivating factors mentioned by the participants such as helping children, the possibility to earn a profitable income, access to medicines, increasing awareness about contraception and immunization, and learning about health and hygiene of her own children and neighbors. The same study reported that community health workers believe that if she or anyone in the village became ill, there would be an advantage in knowing all the health information and to earn a name and fame if she gave treatments for such illnesses; this was considered an additional motivating factor [8, 15]. Further, the findings of this study were consistent with those of a study in Bangladesh, which reported that 86% of VCHWs peruse to volunteer for recognition derived from being needed by the community and the expectation of getting a better job due to the experience gained as a VCHW (86%) were the prominent reasons to continue as VCHWs. Additionally, enjoyment associated with the work, support, and encouragement by family and supervisors were mentioned less frequently by community health workers as motivating factors [15].

In this study, 97.5% of the respondents reaffirm that the community recognizes them as its own servants for the betterment of its welfare. However, according to a study presented in the 13th annual American Public Health association conference, 64% of VCHWs were recognized by the community [16]. Therefore, community recognition is important in lifting the motivation of VCHWs.

Most, 98.5% of VCHWs suggested that they should be selected by the community they work with. However, the same study presented in the 13th annual American Public Health Association conference reported that about 44% of VCHWs expressed good will to community participation in the selection of VCHWs [16].

The average minimal working hours with standard deviation suggested by the VCHWs was 2.30 ± 1.2 hours per day. According to a study presented in the 13th American Annual Public Health conference, the working hours per day was one of the most important factors that affect motivation of VCHWs [16]. Additionally, according to a study performed in South Africa, VCHWs will stay motivated if they have spare time to handle household and family matters [17].

In this study, married women were 4 times more likely to stay motivated than separated ones. This finding is in favor of a study performed in Pakistan which reports that married women with children are more likely to be accepted by the community for the reason that the community members they reach with family planning and other health messages will be more trusting and receptive to their messages because they exemplifying themselves when delivering key health messages [18].

VCHWs within the 20 to 36 year old age group were more likely to stay motivated to continue as VCHWs than the older ones (>36 year of age). However, the same study in Bangladesh found that age, education, marital status, household size, household asset holdings, and duration of stay in their first placement site are a single group of factors that had no notable effect on the motivation of VCHWs [19]. Another study done in Ghana found that age was not significantly associated with motivation of any health workers including VCHWs [20]. This difference could be attributed to the fact that age and other socio-demographic characteristics may be less important for VCHWs to sustain volunteerism.

The limitation of this study was that it only represents perceptions of the VCHWs and HEWs. It did not include the reflection of the community, district health office administrators, and other stakeholders. Additionally, the data collection tool was developed for the first time. Consequently, the reliability and validity of the data collection tool was not determined.

## Conclusions

In conclusion, future training was the most common non-financial incentive mentioned by VCHWs followed by reward proposal (certificate). VCHWs who were within the 20 to 36 years old age group, married women, the existence of children under five years old, allowing volunteer withdrawal, and the establishment of a local endowment fund for the community health workers after they left volunteerism were the factors identified to affect volunteerism. For this reason, implementation of comprehensive packages of non-financial incentives like provision of free medical care, hygienic materials, declaration of VCHWs day, and the provision of rewards for VCHWs are important to sustain volunteerism. Additionally, further research is necessary to assess the role of factors such as the existence of children under five years old, marital status, and the community’s perception about non-financial incentives and motivating factors for VCHWs to sustain volunteerism.

## Abbreviations

CI: Confidence intervals
HEWs: Health Extension Workers
VCHWs: Volunteer community health workers.
